# Genome-Wide Association Studies Based on Equine Joint Angle Measurements Reveal New QTL Affecting the Conformation of Horses

**DOI:** 10.3390/genes10050370

**Published:** 2019-05-14

**Authors:** Annik Imogen Gmel, Thomas Druml, Rudolf von Niederhäusern, Tosso Leeb, Markus Neuditschko

**Affiliations:** 1 Agroscope—Swiss National Stud Farm, Les Longs-Prés, 1580 Avenches, Switzerland; ruedi.vonniederhaeusern@agroscope.admin.ch (R.v.N.); markus.neuditschko@agroscope.admin.ch (M.N.); 2 Institute of Genetics, Vetsuisse Faculty, University of Bern, Bremgartenstrasse 109a, 3012 Bern, Switzerland; tosso.leeb@vetsuisse.unibe.ch; 3 Institute of Animal Breeding and Genetics, Veterinary University Vienna, Veterinärplatz 1, A-1210 Vienna, Austria; thomas.druml@vetmeduni.ac.at

**Keywords:** GWAS, equus caballus, development, growth, carpus, poll, elbow, stifle

## Abstract

The evaluation of conformation traits is an important part of selection for breeding stallions and mares. Some of these judged conformation traits involve joint angles that are associated with performance, health, and longevity. To improve our understanding of the genetic background of joint angles in horses, we have objectively measured the angles of the poll, elbow, carpal, fetlock (front and hind), hip, stifle, and hock joints based on one photograph of each of the 300 Franches-Montagnes (FM) and 224 Lipizzan (LIP) horses. After quality control, genome-wide association studies (GWASs) for these traits were performed on 495 horses, using 374,070 genome-wide single nucleotide polymorphisms (SNPs) in a mixed-effect model. We identified two significant quantitative trait loci (QTL) for the poll angle on ECA28 (*p* = 1.36 × 10^−7^), 50 kb downstream of the *ALX1* gene, involved in cranial morphology, and for the elbow joint on ECA29 (*p* = 1.69 × 10^−7^), 49 kb downstream of the *RSU1* gene, and 75 kb upstream of the *PTER* gene. Both genes are associated with bone mineral density in humans. Furthermore, we identified other suggestive QTL associated with the stifle joint on ECA8 (*p* = 3.10 × 10^−7^); the poll on ECA1 (*p* = 6.83 × 10^−7^); the fetlock joint of the hind limb on ECA27 (*p* = 5.42 × 10^−7^); and the carpal joint angle on ECA3 (*p* = 6.24 × 10^−7^), ECA4 (*p* = 6.07 × 10^−7^), and ECA7 (*p* = 8.83 × 10^−7^). The application of angular measurements in genetic studies may increase our understanding of the underlying genetic effects of important traits in equine breeding.

## 1. Introduction

Conformation, the overall morphology of an animal, is an important selection criterion in horse breeding [1]. It reflects the general appearance of an animal and is associated with locomotor health and sports performance [2]. Conformation is comprised of many traits that describe specific parts of the body (e.g., head and neck, forehand, chest, back, or hindquarters), the distal limb morphology (carpal, fetlock, stifle, and hock joints), or the overall proportions of the animal (e.g., type and harmony) [3,4,5]. The latter are often related to aesthetics, but are also breed specific [6]. Traits such as the slope or inclination of the shoulder and croup, the elbow, carpal, stifle, hock, fetlock joint (pastern) angles, and the shape and angulation of the hoof, have been associated with performance [7,8], longevity [9,10,11,12] or lameness prevalence [13,14] across different breeds and disciplines. Many conformation traits are routinely evaluated during breeding evaluation contests (mare performance tests, field tests, station tests for breeding stallions, etc.) and have been used in breeding value estimation [15,16,17,18,19] and/or genomic analyses, such as genome-wide association studies (GWASs) [20,21]. The heritability (h^2^) for conformation traits mostly ranges between 0.10 and 0.50 [19]. The highest heritability is usually attributed to the height at the withers, and was reported to range from 0.27 [22] to 0.89 [23,24]. Coincidentally, the height at the withers is one of the few routinely measured objective traits in breeding evaluations, while most other traits are either judged based on the breeding optimum, or assessed on a linear scale within each breed [19]. 

Despite conformation traits being widely applied to select breeding individuals and being moderately heritable, the underlying gene variants affecting the morphology of horses are not well known. Several genome-wide association studies have identified quantitative trait loci (QTL) and gene candidates associated with the height at the withers in Franches-Montagnes (FM) horses [20], Quarter Horses [25] and Tennessee Walking Horses [26]. However, within the same studies, other conformation traits, such as the traits describing the distal limb, have not shown any significant association to other regions within the equine genome. In the Franches-Montagnes breed, two different GWASs using the same 1077 individuals and phenotypes, once with the 50K single nucleotide polymorphism (SNP) panel [20] and once with sequence derived genotypes [21], could only confirm the QTL (especially for the height at the withers) from the first study. No new loci were significantly associated with the other conformation traits included in both studies, although several, such as neck muscling, were suggestively associated (*p* < 10^−6^) [21]. This strongly suggests that increased SNP densities are not sufficient to study the genetic variants of the available conformation traits derived from linear description. The data derived from linear description does not seem to account for the complexity of the conformational phenotypes [21]. In practice the linear scale is often not fully used and skewed towards the perceived optimum [15,17]. In some cases, there is little agreement on the optimum between judges [27]. Several conformation traits measured on photographs of the horse, such as the shoulder joint angle or hip joint angle, have shown no relation to the linearly described equivalents of shoulder incline or croup incline [28]. In addition, linear scales are specific to each breeding population, either in their biological range or in the applied scale (1 to 40, 1 to 10, a to i, etc.) [19], and therefore, it is difficult to compare the conformation of different breeds, and restricts the opportunity to increase the sample size for genetic studies.

Recently, a new phenotyping method has been proposed for assessing equine conformation based on photographs. The horse shape space model extracts the shape data from standardized photographs of horses using geometric morphometrics [29]. It consists of a total of 246 landmarks and semi-landmarks tracing the outline of the horse, as well as the specific landmarks of the distal limb. These outlines are normalized using a generalized Procrustes analysis, and the main variation in shape is explained by extracting the relative warp scores (the principal components of the partial warp matrix). Unfavorably for genetic studies, the relative warp scores are dependent on the sample, and are therefore not stable measures. It is, however, also possible to analyze the angular measurements directly from the horse shape space model [28]. Because angles do not depend on size, there is no bias due to the sampling method, as the data does not need to be normalized before extracting the angle measurements. In a previous study, the majority of angular measurements were highly reproducible and consistent within the same animal over different photographs, except for the angles of the shoulder and elbow [28]. The effect of the posture on the joint angles was reduced by creating a classification system of photographs, and correcting the joint angles for relevant posture variables in different statistical models [28]. 

The aims of the study were to compare the morphometric angular measurements of two horse breeds, FM and Lipizzan (LIP), by applying the horse shape space model on photographs, and using the shape-derived joint angles in a GWAS to identify the QTL associated with conformation traits in horses.

## 2. Materials and Methods

### 2.1. Animals

In total, 524 horses were included in this study—300 FM and 224 LIP horses. The 300 Franches-Montagnes horses were all stallions, which were presented at the station test at the Swiss National Stud Farm (SNSF). The stallions were born between 1975 and 2015 (median of 2003), and photographs from the stallion catalogues were preserved in an electronic archive at the SNSF. The DNA samples were routinely isolated from blood samples or sperm doses under permit VD2227.2, building a bio archive curated by the University of Bern and the SNSF of the breeding horse population in Switzerland. The genomic DNA was isolated from the EDTA blood samples using the Maxwell RSC Whole Blood DNA Kit and the Maxwell RSC Instrument (Promega). The Lipizzan sample regrouped photographs and DNA from 224 breeding animals (125 stallions and 99 mares, born between 1987 and 2013; median of 2005) were collected in the studs of Piber (Austria), Đakovo (Croatia), Topol’čianky (Slovakia), and Szilvasvárad (Hungary), during the years 2014 to 2017, following national rules and regulations. 

### 2.2. Phenotyping

#### 2.2.1. Photograph Selection

Each horse was phenotyped based on one unique photograph. The horses were placed in an open posture, as described in the literature [28,29]. Because of the expected variations in the posture, given the difficulties in measuring live animals, the posture of each horse was classified based on the following criteria: head height, head position in relation to the camera, front limb position, hind limb position, body alignment to the camera, and tail carriage, as described in the literature [28]. The year of birth of the horse and the age of the horse in the photograph (if available) was also recorded. The year of birth was divided into four categories (before 1990, 1990 to 1999, 2000 to 2009, and 2010 and younger). This was necessary, particularly for the FM sample, as the photographs were derived from the electronic archive at SNSF, where the individual and its birth year were known, while the date of the photograph was not always available. The age of the horse was also divided into five categories (three to four, five to eight, nine to sixteen, over sixteen years old, and unknown).

#### 2.2.2. Horse Shape Data

**Figure 1:**
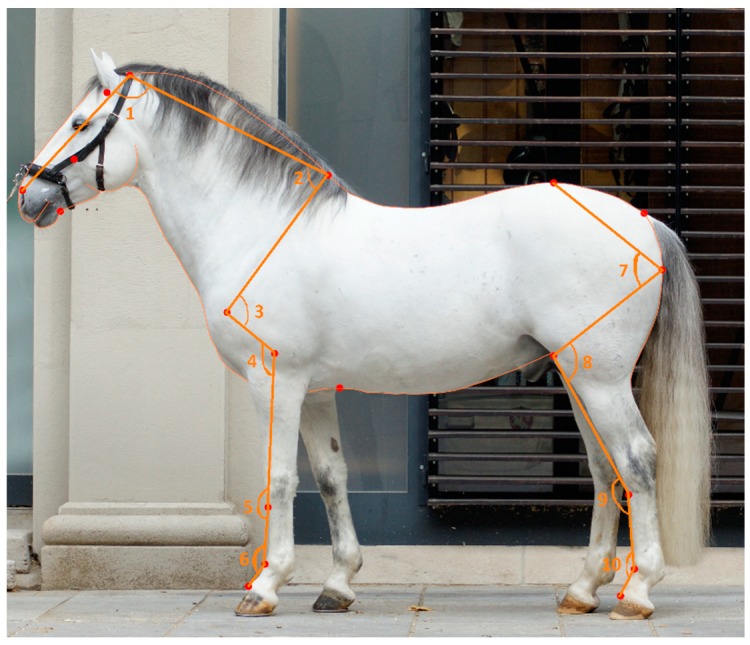
Horse shape space model of a Lipizzan horse photograph, including the following joint angles: (1) poll, (2) neck–shoulder blade, (3) shoulder joint, (4) elbow joint, (5) carpal joint, (6) fetlock joint of the forelimb, (7) hip joint, (8) stifle joint, (9) hock joint, and (10) fetlock joint of the hind limb.

We extracted the shape data of the horses from the photographs using the horse shape space, which is composed of the outline of a horse and 31 additional somatometric landmarks [29]. The semi-landmarks from the curves were placed at equal distances within each curve, and were transformed to landmarks using the computer programmes tpsDig2 and tpsUtil [30,31]. Out of the full shape (246 landmarks, i.e., 31 somatometric and 215 semi-landmarks), we calculated the angles for the available joints, namely: the poll, neck–shoulder blade, shoulder joint, elbow joint, carpal joint, fetlock joint of the forelimb, hip joint, stifle joint, hock, and fetlock joint of the hind limb angles (Figure 1) [28]. The joint angles are independent from the size and orientation of the horses, and were extracted from the raw coordinates using basic trigonometry, as described in the literature [28]. 

#### 2.2.3. Phenotype Concordance

The Franches-Montagnes horses were digitized thrice by the first digitizer (author A.I.G.), and the coordinates were averaged to yield one set of shape data per horse. The Lipizzan horses were digitized once by the second digitizer (author T.D.). To qualify the differences in the shape data that are due to digitizer reproducibility, 20 LIP horses were randomly selected and digitized by A.I.G. The reproducibility of the angular measurements of these horses between T.D. and A.I.G. was calculated with an intraclass correlation coefficient (ICC) in R [32], to decide whether the datasets could be analysed together. We also used the ICC to select the angles with a fair reproducibility (ICC >0.40) [33], whereas the traits with a lower reproducibility were not considered for further analyses. 

#### 2.2.4. Quality Control of the Phenotype

To account for any residual errors and extreme positions in the digitizing process, we filtered the whole shape dataset by removing the individuals for which the Procrustes distance of the shape ranged above the upper quartile of the mean.

### 2.3. Genetic Analyses

#### 2.3.1. Franches-Montagnes Horses

The genetic data of the Franches-Montagnes horses originated from three different sources, namely, previously described imputed 50K SNP data (135 stallions) [34], whole genome sequence data (12 stallions) [34,35], and 670K high density (HD) SNP data (137 stallions), performed by GeneSeek/Neogen on the Affymetrix equine 670K SNP array containing 670,796 evenly distributed genome-wide markers. After the phenotypic filtering, 284 out of 300 genotyped FM stallions were used in the genetic analysis. 

#### 2.3.2. Lipizzan Horses

The genotyping of all of the Lipizzan horses was performed by GeneSeek/Neogen on the Affymetrix equine 670K SNP array. After the phenotypic filtering, 211 (93 mares and 118 stallions) out of the 224 genotyped LIP horses were used in the genetic analysis. 

#### 2.3.3. Merging the Genetic Data Sets (Franches-Montagnes and Lipizzan Horses)

We used PLINK v1.07 software [36,37] to combine the data from the different genotyping sources in the FM and LIP horses (sequence, 50K imputed to sequence, Affymetrix 670K) by extracting the SNPs that were common to all of the datasets (*n* = 514,134 SNPs). We included only the SNPs positioned on the autosomes (*n* = 492,072 SNPs). Furthermore, we removed SNPs with minor allele frequencies (MAF) below 5%, a SNP genotyping rate below 90%, and using the Hardy–Weinberg equilibrium (HWE) with *p* < 0.0001 [20], resulting in 374,070 SNPs for the final analysis. To investigate the population structure of the two breeds, we calculated an identical by state (IBS) derived relationship matrix using PLINK v1.07 software [36,37], followed by a principal component analysis (PCA). 

### 2.4. Genome-Wide Association Studies

Genome-wide association studies were performed on the angle of the poll, elbow joint, carpal joint, fetlock joint of the forelimb, hip joint, stifle joint, hock joint, and fetlock joint of the hind limb using a mixed model approach in the R-package GenABEL [38]. We used a polygenic model approach (*polygenic_hglm)* implemented in GenABEL to account for the population stratification in our sample [39,40]. We first estimated a full model with the birth year (to account for the particularities of the FM sample), the stud farm (to account for the breed and the provenance of each LIP sample, as all FM samples originated from one stud farm), the age category, the sex, and all of the posture variables (head height, head in relation to the camera, position of front limb, hind limb, body alignment to the camera, and tail position) as fixed effects. Using the *summary* function, we identified which fixed effects had a significant effect on the GWAS (i.e., a *p*-value < 0.05). We then excluded the non-significant fixed effects for the final polygenic model, calculated using *polygenic_hgml,* and extracted the significance of each SNP using *mmscore* [41]. We visualised the results using Manhattan plots and considered a *p*-value of 10^-6^ as the threshold for suggestive associations (blue line), as used in the literature [21]. We determined the significance threshold for the effective number of independent loci by pruning the 374,070 SNPs included in the GWAS for linkage disequilibrium (LD) [42], using a 50-kb sliding window size, a 5-kb window step size, and an r^2^ exclusion threshold of 0.5. After the LD pruning, the *p*-value of 0.05 was divided by 187,925 independent SNPs (p_Ind_ < 2.66 × 10^−7^; red line). We also examined the quantile–quantile (Q–Q)-plots for the inflation of small *p*-values, hinting at false positive association signals. Finally, we estimated the genome-wide h^2^ of each angle using GCTA [43]. 

### 2.5. Functional Annotation

We investigated which genes are located near each significant/suggestive QTL by using the NCBI Genome Data Viewer [44], based on the EquCab 2.0 reference genome assembly and Annotation Release 102. The positions of the QTL and the candidate genes within the text refer to the EquCab 2.0 reference genome assembly. The correspondence to the EquCab 3.0 coordinates is also shown in the results section. We evaluated the genes situated approximately 200 kb up- and down-stream of the significant or suggestive QTL(listed in Table S1). We report that the QTL close to the genes have a biological function in the morphology, development, bone metabolism, and bone related disease incidence in human or other animal models, including horses.

## 3. Results

### 3.1. Phenotypic Analyses

The mean, standard deviations, and genome-wide heritability of the angular measurements are presented in Table 1. The heritability ranged between 0.22 and 0.58. We used an intraclass correlation coefficient calculating the intra-specimen variance against the inter-specimen variance to estimate the reproducibility between the two digitisers on a subset of 20 LIP horses. Half of the joint angles (poll, stifle joint, hock joint, and fetlock joint both in the fore and hind limb) showed a good to excellent reproducibility (>0.60, Table 1) [33]. Out of 10 angles, only two (neck–shoulder blade and shoulder joint) did not meet the cut-off value for inclusion in GWAS fixed at an ICC >0.40. Consequently, the neck–shoulder blade and shoulder joint angles were excluded from the subsequent GWAS, while the remaining eight angles were considered reproducible between the two digitisers, and therefore comparable between the two breeds. 

The visualization of the mean of the 20 LIP horses phenotyped by A.I.G. and T.D. (Figure S1) highlights the difference in the placement of the point of shoulder and the fetlock landmarks. This had an effect on the reproducibility of the joint angle measurements, especially on the neck–shoulder blade and shoulder joint angles, and a lesser effect on the elbow and carpal joint angles.

### 3.2. Population Structure

The population structure of the two horse breeds was ascertained with a PCA scatter plot (Figure S2). The first principal component (PC1), accounting for 56% of the variance, clearly separates the horses into two distinct population clusters (FM and LIP), while the second principal component (PC2), accounting for 4% of the variance, further differentiates the LIP horses according to the sample origin.

### 3.3. Genome-Wide Association Studies for Joint Angle Measurements

The summary function of the full *polygenic_hgml* model containing all of the covariates (posture, age, sex, and stud farm) revealed significant (*p* < 0.05) effects of covariates specific to each angle (Table 2, model summaries in File S1 for a detailed description). Thereafter, we conducted GWAS on eight joint angle measurements (poll, elbow joint, carpal joint, hip joint, stifle joint, hock joint, and fetlock joints of the fore and of the hind limb), and accounted for their respective significant covariates. Using *mmscore*, we found significant or highly suggestive associations for the angles of the carpal joint, the elbow joint, the poll, the stifle joint and the fetlock joint of the hind limb with visible effect sizes, which are described hereafter (summary statistics in Table 3). All of the other GWAS results are presented in Table S2. 

#### 3.3.1. Poll Angle Associations

**Figure 2:**
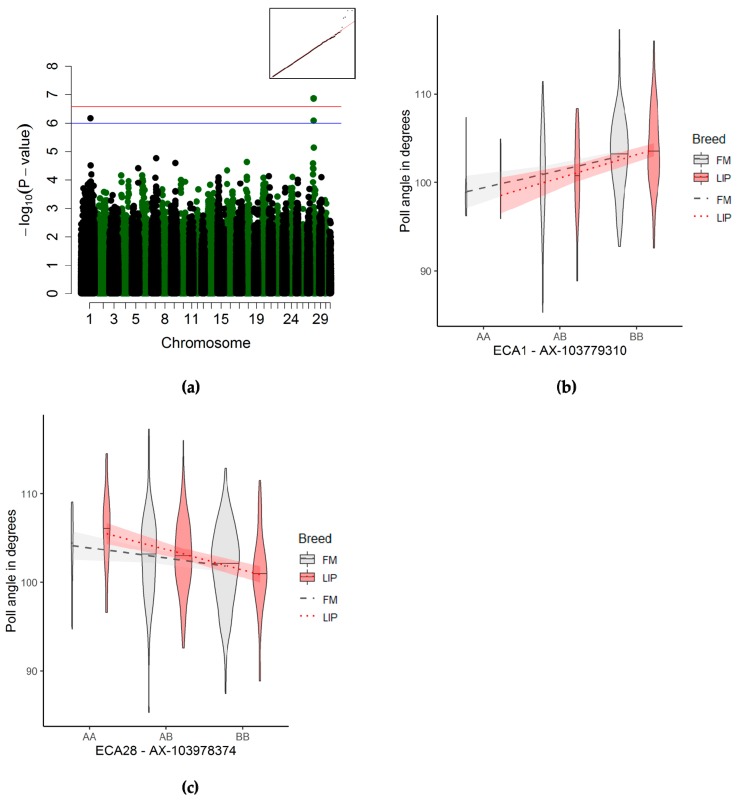
Genome-wide association study (GWAS) for the poll angle (*n* = 495). (**a**) Manhattan plot (blue line) representing the suggestive significance threshold (*p* < 10^−6^) and (red line) the significance threshold corrected for the effectively independent single nucleotide polymorphisms (SNPs) (p_Ind_ <2.66 × 10^−7^). The inset on the right-hand corner shows the quantile–quantile (Q–Q) plot with the observed plotted against the expected *p*-value. (**b**,**c**) Violin plots and trend lines (standard error in translucent) representing the genotype effect of the SNP on ECA1 (b) and ECA28 (c) on the poll angle. The red colour (and dotted line) represent the Lipizzan (LIP) sample; the grey colour (and dashed line) represents the Franches-Montagnes (FM) sample. The width of the violin plot represents the number of animals.

The poll angle was significantly associated with one QTL on ECA28, 113 kb downstream of the *LRRIQ1* gene and 50 kb downstream of the *ALX1* gene (Figure 2a and Table 3). Additionally, this angle was suggestively associated with another QTL on ECA1, 95 kb downstream of the gene *CORO2B*, and 162 resp. 182 kb upstream of the genes *ITGA11* and *FEM1B* (Table S1). For the QTL on ECA1, the horses homozygous for the reference allele had a smaller poll angle than the alternate allele (Figure 2b), while the trend was reversed for the QTL on ECA28 (Figure 2c). 

#### 3.3.2. Elbow Joint Angle Association

**Figure 3:**
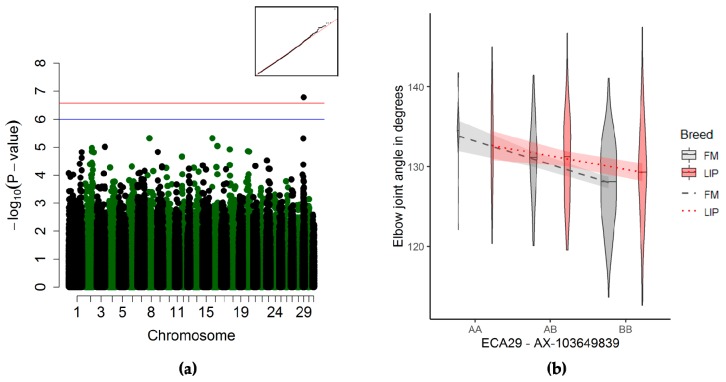
Genome-wide association study (GWAS) for the elbow joint angle (*n* = 495). (**a**) Manhattan plot and Q–Q-plot (inset) with the blue line representing the suggestive significance threshold (*p* < 10^−6^) and the red line representing the significance threshold (p_Ind_ <2.66 × 10^−7^). (**b**) Violin plot and trend lines (standard error in translucent) representing the genotype effect of the significant SNP on ECA29 on the elbow joint angle.

The significant quantitative trait locus for the elbow joint angle (Table 3 and Figure 3a) was surrounded by three genes, namely: *RSU1* (49 kb upstream of the QTL), *PTER* (75 kb downstream of the QTL), and *C1QL3* (16 kb downstream of the QTL) (Table S1). Horses homozygous for the alternate allele presented with a smaller elbow joint angle than horses homozygous for the reference allele (Figure 3b).

#### 3.3.3. Stifle Joint Angle Association

**Figure 4:**
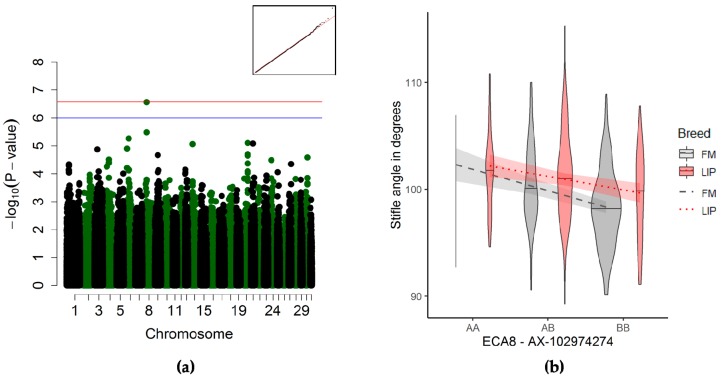
Genome-wide association study (GWAS) for the stifle joint angle (*n* = 495). (**a**) Manhattan plot and Q–Q-plot (inset) (blue line) representing the suggestive significance threshold (*p* < 10^−6^) and (red line) the significance threshold (p_Ind_ <2.66 × 10^−7^). (**b**) Violin plot and trend lines (standard error in translucent) representing the genotype effect of the significant SNP on ECA29 on the elbow joint angle.

The suggestive quantitative trait locus on ECA8 affecting the stifle angle (Figure 4a and Table 3) was surrounded by 11 genes, whose functions are described in Table S1. The horses homozygous for the reference allele had larger stifle angles than the homozygous alternate allele (Figure 4b). 

#### 3.3.4. Fetlock Joint Angle of the Hind Limb Association

**Figure 5:**
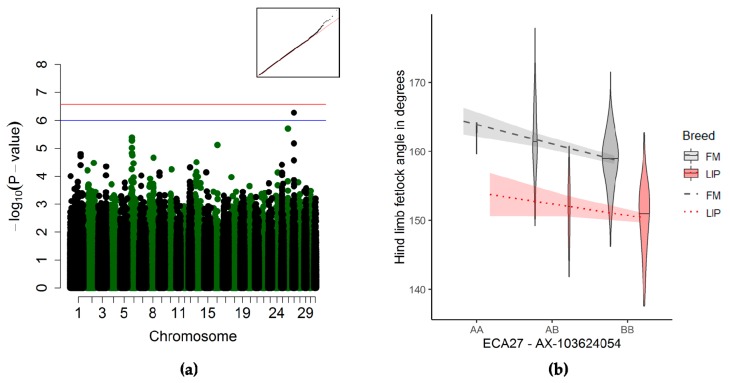
Genome-wide association study (GWAS) for the fetlock joint angle of the hind limb (*n* = 495). (**a**) Manhattan plot and Q–Q-plot (inset) (blue line) representing the suggestive significance threshold (*p* < 10^−6^) and (red line) the significance threshold (p_Ind_ <2.66 × 10^−7^). (**b**) Violin plot and trend lines (standard error in translucent) representing the genotype effect of the significant SNP on ECA27 on the elbow joint angle.

The fetlock joint angle of the hind limb was suggestively associated with a QTL on ECA27 (Figure 5a and Table 3). The quantitative trait locus mapped to no known gene—the nearest genes were mapped over 500 kb up- or down-stream of the QTL. Horses homozygous for the reference allele (only two FM and three LIP horses) had a larger fetlock angle than the horses with at least one alternate allele (Figure 5b). 

#### 3.3.5. Carpal Joint Angle Associations

**Figure 6:**
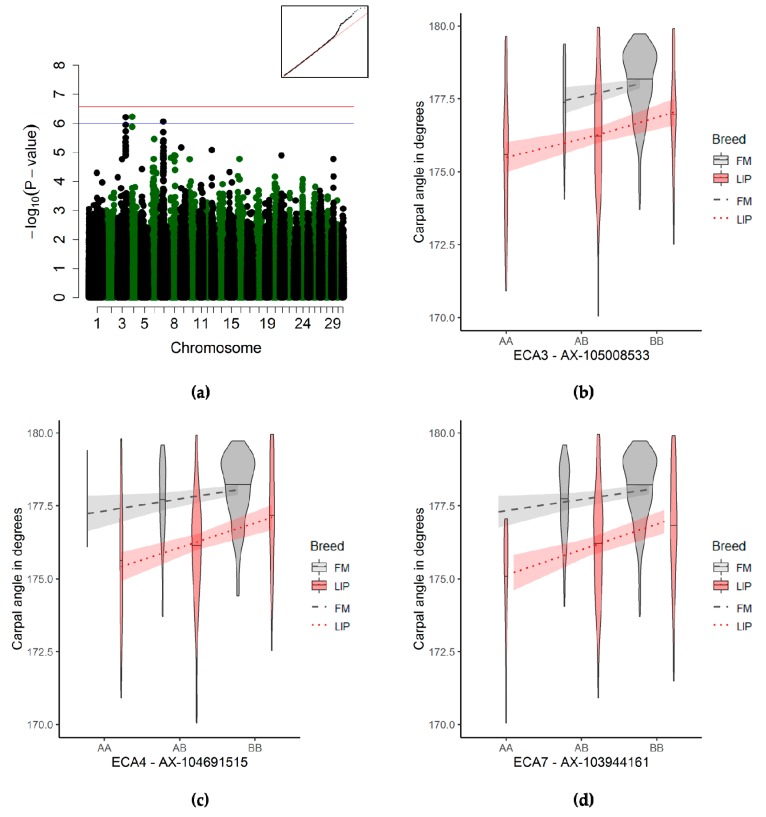
Genome-wide association study (GWAS) for the carpal joint angle (*n* = 495). (**a**) Manhattan plot and Q–Q-plot (inset) (blue line) representing the suggestive significance threshold (*p* < 10^−6^) and the (red line) the significance threshold (*p* < 2.66 × 10^− 7^). (**b**–**d**) Violin plots and trend lines (standard error in translucent) representing the genotype effect of the significant SNP on ECA3 (b), ECA4 (c), and ECA7 (d) on the carpal joint angle.

The carpal angle showed three suggestive genome-wide associations (Figure 6a and Table 3). The best-associated QTL on the ECA4 was 281 kb downstream of the *CALCR* gene (Table S1). The second-best QTL for the carpal angle was located 329–370 kb downstream of the *LCORL/NCAPG* genes on ECA3 (Table S1). A third QTL was highly suggestive and located 302 kb downstream of the *NCAPD3* gene (Table S1). The horses homozygous for the reference allele have a smaller carpal angle than the horses with at least one alternate allele of the loci (Figure 6b,c,d).

## 4. Discussion

### 4.1. Phenotypes

The phenotype concordance was sufficiently high to pool the phenotypes digitised by two different persons together. There were only two angles that had to be excluded because of a low reproducibility. These angles had already shown problematic reproducibility and consistency in a previous study because of the placement of the point of the shoulder [28]. We saw slight differences in the placement of the landmarks not only at the point of the shoulder, but also at the fetlock, which had an impact on the reproducibility between the two digitisers. This may also have an effect on the overall differences between the two breeds, as different people digitised the two breeds. Other angles with a lower reproducibility can be explained by a lower variance within the trait, as the ICC is a measure of intra- versus inter-individual variance. This is the case for a lower ICC in the carpal angle, ranging between 170.00 and 179.90 degrees. Joint angles with a high variance and high reproducibility are more reliable, for example, the poll (85.35 to 117.29 degrees; ICC = 0.92) or the fetlock joint angle of the hind limb (137.60 to 177.90 degrees; ICC = 0.81). 

The genome-wide heritability of the different joint angle measurements ranged between 0.22 and 0.58. The heritability was in the same range as most of the conformation traits described on a linear scale. The sample size of this study, however, was smaller than those that previously reported h^2^ for the conformation traits [19]. More horses should be pheno- and geno-typed to reassess the h^2^ of the measures we proposed here.

Because the size and the complexity of the sample, consisting of two unrelated breeds, we elected to do a preselection of photographs and filtered out extreme shapes using the mean Procrustes distance as a reference. We opted to systematically and neutrally filter out the most disruptive elements, but keep as many animals as possible in the analysis. For this study, we carefully evaluated each GWAS model to include the covariants with the most significant outcome on the GWAS. This has allowed us to correct for external non-genetic effects, while maintaining a higher sample size of horses. The posture of the horse in the photograph had a strong effect on the angle measures, as previously shown [28]. 

### 4.2. Genome-Wide Association Studies

#### 4.2.1. Identification and Interpretation of Relevant Quantitative Trait Loci

We used joint angle measurements for two independent breeds to maximise the sample size within our GWAS. To avoid breed specific bias, we corrected for relatedness by using a kinship matrix in our GWAS models, and included the stud farm as a fixed environmental effect wherever the model summary suggested a significant association of the stud farm on the outcome (only for the fetlock angle of the hind limb). We carefully analysed the effect of the genotype on the phenotype for each breed, so as to avoid reporting associations that result only from differences between the breeds. For all of our reported associations, the trend lines were unidirectional and affected both breeds in a similar manner. 

#### 4.2.2. Poll Angle

For the poll angle, the significant QTL on ECA28 was situated within a long stretch of DNA behind the actual gene void of any other gene (i.e., a “gene desert”). It was however 50 kb downstream of the *ALX1* gene, a homeobox gene critical to the development of the heads and spines in mammals [45]. Mutations in this gene have been reported to cause cleft palate and/or lip (CL/P) in humans [46,47]. A phenotype closely related to the poll angle we studied here, a specific occipitoatlantoaxial malformation, was found, in one case study of an Arabian horse, to be likely caused by a deletion near the *HOXD3* gene, another homeobox gene. The diseased horse presented with, among other symptoms, an abnormal head position, extended neck posture (i.e., large poll angle), and visible abnormalities in the cervical spine. Two other individuals presented with similar phenotypes, but did not have this particular likely causal deletion in their genotype. The abnormal head position and extended neck seem to reflect an extreme case of the phenotypic variation shown in our horse sample. In our study, two loci were associated with this angle, and there may be more QTL with similar effects having an influence on head–neck morphology, as was also suggested in the literature [48] for occipitoatlantoaxial malformations. 

In the domestic horse, several studies have reported on runs of homozygosity (ROH) in regions containing homeobox genes. Runs of homozygosity islands containing the genes *HOXB1, HOXB2, HOXB3, HOXB5, HOXB6, HOXB7, HOXB8,* and *HOXB13* were identified in the Lipizzan horse breed [49], and were reported in the Posavje horse, a native breed of Eastern Europe [50]. A recent study reported a private ROH variant in four Hannoverian horses containing the gene *ALX4* [51], which belongs to the same family as *ALX1,* and shares most of its functions [45,52]. Genomic changes in the neural crest cells are thought to be one of the primary drivers of behavioral, physiological, and morphological changes during the domestication of animals [53]. Among the characteristics changed under domestication were also changes in the craniofacial morphology (e.g., shorter snouts, smaller teeth, and larger eyes) [53]. This “neural crest” theory was recently supported by a study using ancient horse DNA in comparison to current horse breeds, showing an enrichment of genes involved in neural crest development and morphology in domesticated horses [54]. We could not identify any obvious candidate genes with known functions in the bone morphology or metabolism near the QTL on ECA1.

#### 4.2.3. Elbow Joint Angle

The significant quantitative trait locus on ECA29 associated with the elbow angle was located between the *RSU1* and *PTER* genes. The gene *RSU1,* 49 kb upstream of the QTL, is associated with body height and bone mineral density in humans. The gene *PTER*, 75 kb downstream of the QTL, is also associated with an increased risk of bone fractures and osteoporosis in humans [55]. In horses, lameness due to elbow injury is rare [56]. However, stress fractures in the humerus (the bone between the shoulder and elbow point we measured) have been reported to be relatively common in racehorses coming back into racing after a break in exercise regime [57]. Fractures within the elbow joint can occur in young horses, and are often caused by fragile growth plates (epiphyseal fractures). Other fractures of the elbow are less frequent, and are caused by trauma (falls or kicks from another horse) [56,58]. Because of the overall low prevalence of elbow-related lameness and strong environmental effects (trauma and exercise regime), the link between the QTL we reported here and the elbow angle needs to be further investigated. 

#### 4.2.4. Stifle Joint Angle

For the stifle joint angle, the suggestive QTL on ECA8 showed no association with the genes involved in the bone morphology or metabolism, except the *RASAL1* gene, 142 kb downstream of the QTL, linked to ossifying fibroma and other benign bone growths in humans [59,60]. All 11 genes within 200 kb up- or down-stream of this QTL were linked to body mass index in humans (Table S1). One gene, *LHX5*, 124 kb upstream of the QTL, is also a homeobox gene associated with neural crest differentiation and forebrain development [61,62]. However, several other genes lie between the identified QTL and this particular homeobox gene, and the potential effects on the phenotype are unclear.

#### 4.2.5. Fetlock Joint Angle of the Hind Limb

The locus on ECA27 suggestively associated with the fetlock angle of the hind limb was mapped to another gene desert. The closest genes were at least 500 kb up- or down-stream of the suggestive QTL. Interestingly, the 827 kb upstream flanking gene was *FRG1*, involved in muscular development and associated with fascioscapulohumeral muscular dystrophy 1 in humans [63]. The link between a gene affecting the structures of the forelimb (scapula and humerus) and the fetlock joint angle of the hind limb is unclear. 

#### 4.2.6. Carpal Joint Angle

Three QTL were suggestively associated with the carpal joint angle. The trait back-at-the-knee, which is the closest approximation to our small carpal angle, was associated with uni- and bi-lateral carpal joint arthritis in a study of Norwegian trotters [64], and has shown a very high heritability (h^2^ = 0.66) in a study including a total of 3916 Thoroughbred racehorses in the United Kingdom [11]. Therefore, the genetic architecture of this conformation trait appears favourable for uncovering associated genetic regions. From our results, we also see that there are multiple QTLs having an effect on this phenotype. The best association affecting the carpal joint angle was detected on ECA4, 281 kb from the *CALCR* gene that encodes for the calcitonin receptor. The calcitonin receptor mediates calcium homeostasis, bone mineral density by osteoclast-mediated bone resorption, and its dysfunction is associated with osteoporosis [55]. Carpal fractures are relatively common in racehorses and are an important factor in the decision to retire a horse early [57]. In addition, a higher carpal angle decreases the odds of a carpal fracture in Thoroughbreds [13]. It is therefore possible that the *CALCR* gene is linked to the weakening of the carpal bones in horses with a small carpal angle, increasing the risk of fractures. 

The quantitative trait locus on ECA3 at 105 mb associated with the carpal angle was identified in several equine studies [20,21]. In these studies, the QTL was linked with the *LCORL/NCAPG* gene, which has been associated with height [26,65,66,67]. In our study, the QTL was associated with the carpal joint angle. This would confirm the findings from the authors of [20], who found similar associations with the linearly described trait conformation of legs, which is based, in part, on the visual appraisal of the carpal angle. The window around the third QTL associated with the carpal angle harbours only one potential gene affecting the phenotype. The gene *NCAPD3* (also named *CAPN3*), 302 kb upstream of the QTL has been associated with a short stature, limb hypertonia, and poor overall growth [68,69]. Interestingly, the *NCAPD3* gene may have a functional relation to the *NCAPG* gene, as both genes encode subunits of non-SMC condensin complexes, which have a role in stabilising the chromosomes during mitosis and meiosis [70], and seem closely associated to growth. 

## 5. Conclusions

Using joint angle measurements extracted from the horse shape space model as phenotypes in GWAS, this study identified several new QTL potentially involved in the conformation of horses. Two quantitative trait loci, one for the poll and one for the fetlock joint of the hind limb, were situated in gene deserts and surrounded by important developmental genes (*ALX1* and *FGR1*) involved in musculoskeletal development in mammals. Two quantitative trait loci for the carpal and elbow joint angles were located near the genes associated with osteoporosis in humans (*CALCR* and *RSU1/PTER*, respectively). Two other quantitative trait loci for the carpal joint angle were near the genes associated with height (*LCORL/NCAPG* in horses and *NCAPD3* in humans). Future studies with independent samples should be performed in order to confirm these findings. 

## Figures and Tables

**Table 1:** Mean, standard deviation (SD), minimum, maximum, genome-wide heritability, and standard error (SE) for 495 horses, and the reproducibility (ICC) of the joint angle measurements of 20 Lipizzan horses (LIP).

Joint Angle	Mean	SD	Min	Max	h^2^	SE	ICC (20 LIP)
Poll	102.55	4.70	85.35	117.29	0.38	0.098	0.92
Neck–shoulder blade	81.86	6.56	62.77	99.47	0.42	0.086	0.16
Shoulder joint	94.00	6.61	77.09	114.94	0.42	0.090	0.13
Elbow joint	129.50	5.79	112.60	147.50	0.22	0.076	0.52
Carpal joint	177.30	1.81	170.00	179.90	0.35	0.088	0.49
Fetlock joint of the forelimb	149.10	4.08	136.80	162.00	0.32	0.089	0.79
Hip joint	78.22	3.01	68.80	91.17	0.24	0.092	0.58
Stifle joint	99.76	4.08	89.28	115.26	0.40	0.089	0.90
Hock	152.90	2.30	145.00	159.50	0.25	0.095	0.96
Fetlock joint of the hind limb	155.80	6.43	137.60	177.90	0.58	0.086	0.81

**Table 2:** Summary table of the significance of the covariates in the full polygenic model.

Joint Angle	Stud Farm	Birth Year Category	Age Category	Sex	Head in Relation to Camera	Position of Front Limb	Position of Hind Limb	Body Alignment to the Camera	Tail Position
Poll			**	*	**			*	**
Elbow joint						***		**	
Carpal joint				***					
Fetlock joint of the forelimb				**			*		
Hip joint		**		*			***	***	
Stifle joint			***		*		***	***	
Hock							***		
Fetlock joint of the hind limb	*					***	**		

*= *p*-value < 0.05, ** = *p*-value < 0.01, ***=*p*-value < 0.001.

**Table 3:** Best associations by trait (only markers with *p* < 10^−6^ are reported) for the genome-wide association studies of Franches-Montagnes (FM) and Lipizzan (LIP) joint angle measurements.

Joint Angle	ECA	Position on EquCab2.0	Position on EquCab 3.0	#SNP	SNP with the Lowest *p*-Value	*p*-Value	Number of Genotyped Horses	Allele Frequency		
								AA_FM,LIP_	AB_FM,LIP_	BB_FM,LIP_
Poll	28	12,101,898–12,106,363	13,129,017–13,133,483	3	AX-103978374	1.36 × 10^−07^	495	49_11,38_	197_97,100_	249_176,73_
	1	124,405,158	125,551,151	1	AX-103779310	6.83 × 10^−07^	495	13_8,5_	133_70,63_	349_206,143_
Elbow joint	29	18,799,958	19,878,299	1	AX-103649839	1.69 × 10^−07^	490	37_12,25_	154_69,85_	299_198,101_
Stifle joint	8	19,266,146	21,704,931	1	AX-102974274	2.77 × 10^−07^	473	45_4,41_	186_80,106_	242_178,64_
Fetlock joint of the hind limb	27	22,021,462	22,068,848	1	AX-103624054	5.42 × 10^−07^	495	5_3,2_	102_70,32_	388_211,177_
Carpal joint	4	37,412,203	37,460,405	1	AX-104691515	6.07 × 10^−07^	479	46_3,43_	160_54,106_	273_211,62_
	3	106,128,177	107,955,102	1	AX-105008533	6.24 × 10^−07^	493	39_0,39_	143_28,115_	311_254,57_
	7	42,659,817	43,694,770	1	AX-103944161	8.83 × 10^−07^	495	22_2,20_	186_75,111_	287_207,80_

#SNP—the number of single nucleotide polymorphisms for a specific trait that passed the suggestive *p*-value threshold of 10^-6^. *p*-value—*p*-value of the SNP with the lowest *p*-value per trait and locus corrected for genomic inflation (Pc1df from GenABEL).

## Supplementary Materials

The following are available online at https://www.mdpi.com/2073-4425/10/5/370/s1: Table S1: Summary of genes near the identified QTL and their functions in humans based on the GeneCards database, Figure S1: Differences in the mean shape of 20 Lipizzan horses digitized by A.I.G. (orange) and T.D. (red) by superimposition; Figure S2: Population stratification; File S1: Model evaluation for GWAS; Table S2: Summary table of all GWAS.
