# Supplementary material for: Genome-Wide Association Studies Based on Equine Joint Angle Measurements Reveal New QTL Affecting the Conformation of Horses
Source: Genes (Basel). 2019 May 14;10(5):370. doi: 10.3390/genes10050370 (PMC6562990; doi:10.3390/genes10050370)

Figure S1: Differences in the mean shape of 20 Lipizzan horses digitized by author A.I.G. (orange) and author T.D. (red) by superimposition

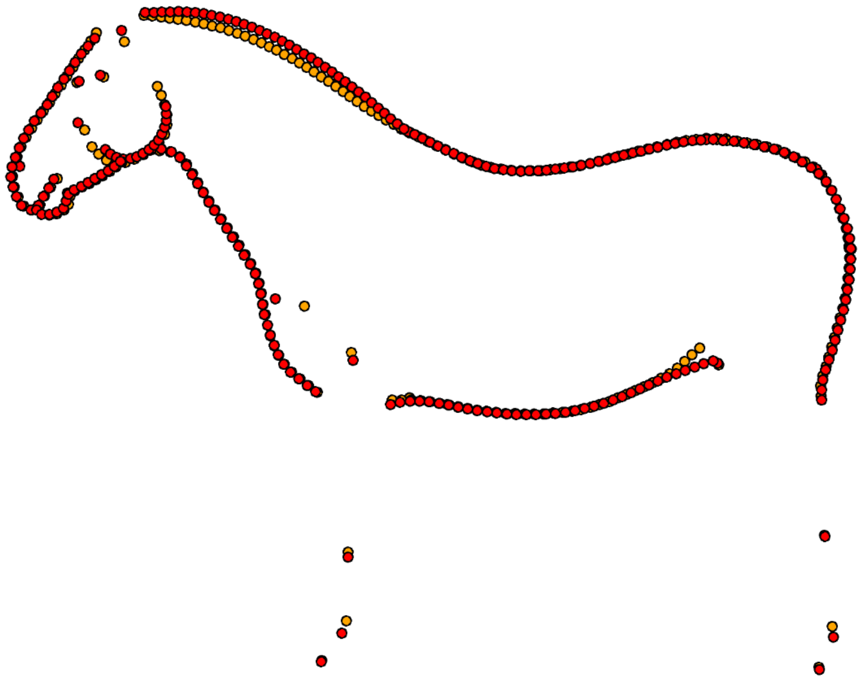

Supplement: Supplementary file 1 [file genes-10-00370-s001.zip › Figure S1.pdf]
