# Supplementary material for: Genome-Wide Association Studies Based on Equine Joint Angle Measurements Reveal New QTL Affecting the Conformation of Horses
Source: Genes (Basel). 2019 May 14;10(5):370. doi: 10.3390/genes10050370 (PMC6562990; doi:10.3390/genes10050370)
Supplement: Supplementary file 1 [file genes-10-00370-s001.zip › Figure S2.pdf]

Figure S2 Population stratification

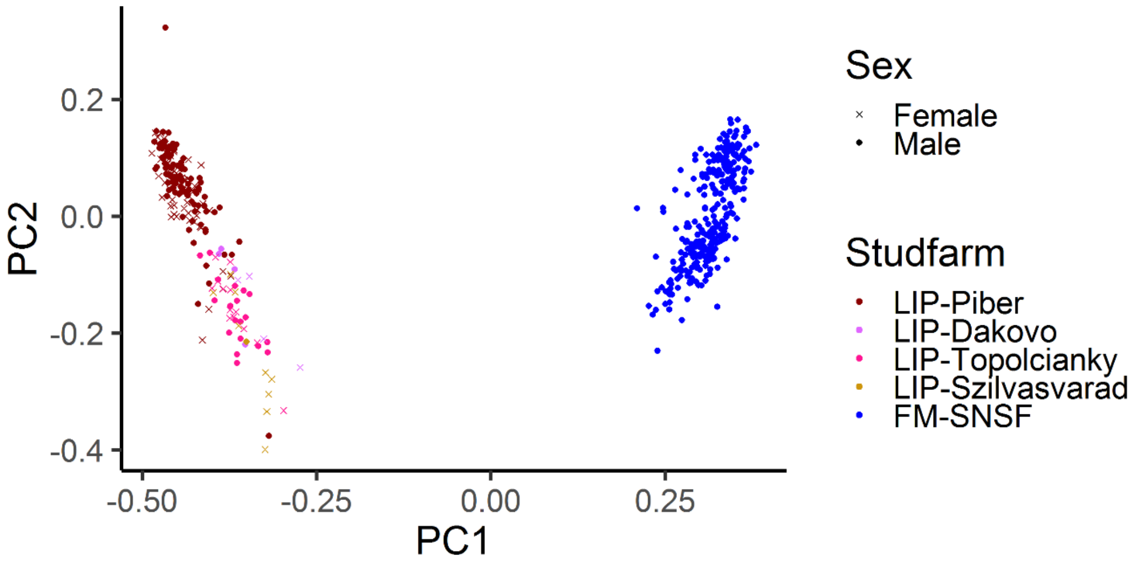

Visualisation of the 495 horses (284 FM and 211 LIP) on the first (PC1) and second (PC2) principal component. The colours of the horses represents the geographical origin, whilst the different symbols reflects the sex of the horses.
