# Supplementary material for: Genome-Wide Association Studies Based on Equine Joint Angle Measurements Reveal New QTL Affecting the Conformation of Horses
Source: Genes (Basel). 2019 May 14;10(5):370. doi: 10.3390/genes10050370 (PMC6562990; doi:10.3390/genes10050370)
Supplement: Supplementary file 1 [file genes-10-00370-s001.zip › File S1.pdf]

File S1 Model evaluation for GWAS

$y_i = \mu + \beta_{i,j} \times (g_i \times kin)$

where g is the genotype of the individual i, kin is the kinship matrix, μ is the grand population mean (intercept) and β_j are the relevant covariates

β_1	Stud farm
β_2	Birth year category
β_3	Age Category
β_4	Sex
β_5	Head height
β_6	Head in relation to the camera
β_7	Position of front limb
β_8	Position of hind limb
β_9	Body alignment to the camera
B_{10}	Tail position

S1.1 Poll angle

Summary of fixed effects estimates

	Estimate	SE	t-value	p-value
Intercept	*104.61906*	*2.48205*	*42.150*	*<0.0001*
Stud farm	-0.02361	0.34505	-0.068	0.94548
Birth year category	-0.45611	0.32852	-1.388	0.16598
**Age category**	**0.63858**	**0.19954**	**3.200**	**0.00151**
**Sex**	**1.28069**	**0.60799**	**2.106**	**0.03594**
Head height	-0.82122	0.58836	-1.396	0.16375
**Head in relation to the camera**	**0.73798**	**0.23912**	**3.086**	**0.00220**
Position of front limb	-0.04052	0.23783	-0.170	0.86483
Position of hind limb	0.20609	0.24673	0.835	0.40417
**Body alignment to the camera**	**-0.65229**	**0.31906**	**-2.044**	**0.04172**
**Tail position**	**-1.72304**	**0.54136**	**-3.183**	**0.00160**

S1.2 Elbow joint angle

Summary of fixed effects estimates

	Estimate	SE	t-value	p-value
Intercept	*141.2325*	*3.0331*	*46.564*	*< 0.0001*
Stud farm	0.4934	0.4135	1.193	0.23358
Birth year category	0.5727	0.4015	1.426	0.15476
Age category	-0.3879	0.2443	-1.588	0.11329
Sex	-1.4567	0.7437	-1.959	0.05098
Head height	-1.3872	0.7206	-1.925	0.05506
Head in relation to the camera	-0.2896	0.293	-0.988	0.3238
**Position of front limb**	**-1.6461**	**0.2911**	**-5.655**	**<0.0001**
Position of hind limb	-0.54	0.3021	-1.787	0.07479
**Body alignment to the camera**	**-1.0562**	**0.3909**	**-2.702**	**0.00725**
Tail position	0.3991	0.6625	0.602	0.54727

S1.3 Carpal joint angle

Summary of fixed effects estimates

	Estimate	SE	t-value	p-value
Intercept	*176.2778*	*0.894609*	*197.045*	*< 0.0001*
Stud farm	0.023023	0.123869	0.186	0.853
Birth year category	-0.00251	0.118413	-0.021	0.983
Age category	0.090519	0.071949	1.258	0.209
**Sex**	**1.0416**	**0.21918**	**4.752**	**< 0.0001**
Head height	-0.04098	0.212162	-0.193	0.847
Head in relation to the camera	-0.15851	0.086236	-1.838	0.067
Position of front limb	0.009347	0.085749	0.109	0.91
Position of hind limb	0.102076	0.088966	1.147	0.252
Body alignment to the camera	0.15116	0.11506	1.314	0.19
Tail position	-0.1521	0.19518	-0.779	0.436

S1.4 Fetlock joint angle of the forelimb

Summary of fixed effects estimates

	Estimate	SE	t-value	p-value
Intercept	*146.8066*	*2.13286*	*68.831*	*< 0.0001*
Stud farm	0.28005	0.29437	0.951	0.34212
Birth year category	-0.23838	0.28232	-0.844	0.3991
Age category	0.15338	0.17158	0.894	0.37204
**Sex**	**1.5692**	**0.52263**	**3.002**	**0.00289**
Head height	0.09664	0.506	0.191	0.84865
Head in relation to the camera	-0.07419	0.20569	-0.361	0.71857
Position of front limb	0.30341	0.20449	1.484	0.14
**Position of hind limb**	**0.53665**	**0.21218**	**2.529**	**0.0119**
Body alignment to the camera	-0.41089	0.27443	-1.497	0.1353
Tail position	-0.18696	0.46544	-0.402	0.68818

S1.5 Hip joint angle

Summary of fixed effects estimates

	Estimate	SE	t-value	p-value
Intercept	*82.63606*	*1.39918*	*59.061*	*< 0.0001*
Stud farm	-0.09943	0.19169	-0.519	0.60431
**Birth year category**	**-0.6051**	**0.18522**	**-3.267**	**0.001202**
Age category	-0.15258	0.11263	-1.355	0.176472
**Sex**	**-0.7404**	**0.34298**	**-2.159**	**0.031593**
Head height	-0.2887	0.33222	-0.869	0.385474
Head in relation to the camera	0.06353	0.13508	0.47	0.63842
Position of front limb	-0.06525	0.13423	-0.486	0.63
**Position of hind limb**	**-1.85089**	**0.13929**	**-13.288**	**< 0.0001**
**Body alignment to the camera**	**0.63432**	**0.1802**	**3.52**	**0.000492**
Tail position	0.5177	0.30549	1.695	0.091088

S1.6 Stifle joint angle

Summary of fixed effects estimates

	Estimate	SE	t-value	p-value
Intercept	*86.3356*	*1.8994*	*45.454*	*< 0.0001*
Stud farm	0.2866	0.2631	1.089	0.276818
Birth year category	0.2539	0.2514	1.01	0.313351
**Age category**	**0.5486**	**0.1528**	**3.592**	**0.000379**
Sex	-0.3919	0.4653	-0.842	0.400278
Head height	0.4822	0.4504	1.07	0.285198
**Head in relation to the camera**	**0.4427**	**0.1831**	**2.418**	**0.016149**
Position of front limb	0.1138	0.1821	0.625	0.53
**Position of hind limb**	**1.9411**	**0.1889**	**10.277**	**<0.0001**
**Body alignment to the camera**	**1.3655**	**0.2443**	**5.59**	**<0.0001**
Tail position	0.6183	0.4144	1.492	0.136622

S1.7 Hock angle

Summary of fixed effects estimates

	Estimate	SE	t-value	p-value
Intercept	*153.6902*	*1.15834*	*132.681*	*< 0.0001*
Stud farm	0.03015	0.15974	0.189	0.8504
Birth year category	0.10144	0.15333	0.662	0.5087
Age category	0.10509	0.09319	1.128	0.2603
Sex	0.48366	0.28385	1.704	0.0894
Head height	-0.0679	0.27483	-0.247	0.805
Head in relation to the camera	0.13307	0.11172	1.191	0.2345
Position of front limb	-0.06313	0.11107	-0.568	0.57
**Position of hind limb**	**-1.08524**	**0.11524**	**-9.417**	**< 0.0001**
Body alignment to the camera	0.24681	0.14906	1.656	0.0987
Tail position	0.1087	0.25279	0.43	0.6675

S1.8 Fetlock joint angle of the hind limb

Summary of fixed effects estimates

	Estimate	SE	t-value	p-value
Intercept	*159.8484*	*2.55466*	*62.571*	*< 0.0001*
**Stud farm**	**0.71303**	**0.35974**	**1.982**	**0.04834**
Birth year category	0.04059	0.33806	0.12	0.9045
Age category	0.22598	0.20512	1.102	0.27144
Sex	1.18081	0.62537	1.888	0.05992
Head height	-0.4693	0.60467	-0.776	0.43826
Head in relation to the camera	-0.25714	0.24564	-1.047	0.29598
**Position of front limb**	**-1.18611**	**0.2445**	**-4.851**	**< 0.0001**
**Position of hind limb**	**-0.73777**	**0.25361**	**-2.909**	**0.00388**
Body alignment to the camera	0.2483	0.32782	0.757	0.44936
Tail position	-0.89216	0.55668	-1.603	0.11001
