# Supplementary material for: Genome-Wide Association Studies Based on Equine Joint Angle Measurements Reveal New QTL Affecting the Conformation of Horses
Source: Genes (Basel). 2019 May 14;10(5):370. doi: 10.3390/genes10050370 (PMC6562990; doi:10.3390/genes10050370)
Supplement: Supplementary file 1 [file genes-10-00370-s001.zip › Table S1.pdf]

Table S1: Summary of all genes near the identified QTL and their functions in humans based on the GeneCards database

Trait	ECA	Genes near the QTL	Location of gene on EquCab2.0	Location of gene on EquCab3.0	Associated human phenotypes or diseases
Poll	1	*CORO2B*	124’181’094 – 124’310’067	125’326’866 – 125’463’477	Diastolic and systolic blood pressure, neuropsychological tests
		ITGA11	124’447’398 – 124’566’837	125’593’167 – 125’711’828	Dupuytren contracture, systolic blood pressure, attention deficit hyperactivity disorder, unipolar depression, serum IgG glycolysation measurement
		FEM1B	124’572’574 – 124’587’241	125’716’242 – 125’733’251	Dupuytren contracture, systolic blood pressure, unipolar depression
	28	*LRRIQ1*	11’806’213 – 11’993’045	12’828’749 – 13’042’786	Synophrys measurement, femoral neck bone mineral density
		ALX1	12’029’878 – 12’051’698	13’056’871 – 13’078’827	Nose morphology measurement, femoral neck bone mineral density, Frontonasal dysplasia 3 (true ocular hypertelorism; broadening of the nasal root; median facial cleft affecting the nose and/or upper lip and palate; unilateral or bilateral clefting of the alae nasi; lack of formation of the nasal tip; anterior cranium bifidum occultum; a V-shaped or widow's peak frontal hairline)
Elbow joint	29	*RSU1*	18’575’605 – 18’751’396	19’648’682 – 19’829’810	Platelet component distribution width, albuminuria, body height, migraine disorder, bone density
		C1QL3	18’809’431 – 18’816’434	19’884’891 – 19’896’116	Adolescent idiopathic scoliosis
		PTER	18’818’433 – 18’874’900	19’896’602 – 19’953’158	Obesity, adolescent idiopathic scoliosis, urate measurement, body mass index, bone fracture, osteoporosis, eye morphology measurement, Waardenburg Syndrome Type II (pigmentary abnormalities of the hair, skin, and eyes, congenital sensorineural hearing loss, and absence of lateral

					displacement of the inner canthus of each eye), Chromosome 16P13.3 Duplication Syndrome (possible symptoms include developmental delay, speech delay, joint abnormalities, characteristic facial features, attention deficit, autism spectrum disorders, and underlying health problems such as heart conditions)
Stifle joint	8	*LHX5*	19'133'640 – 19'142’420	21'571'080 – 21'580’907	Neural crest differentiation, Transcriptional Regulatory Network in Embryonic Stem Cell, body mass index, erosive tooth wear measurement, cerebral amyloid deposition measurement, glomerular filtration rate, chronic kidney disease
		SDSL	19'159'497 – 19'173’479	21'597'005 – 21'610’807	Body mass index, high density lipoprotein cholesterol measurement, parasagittal meningioma, cerebral convexity meningioma
		SDS	19'181'653 – 19'190’469	21'619'157 – 21'627’979	Salla disease (hypotonia, cerebellar ataxia, and mental retardation), heel bone mineral density, abnormality of the foot
		SLC8B1	19'233'776 – 19'251’178	21'664'510 – 21'690’376	Body mass index, Plays a central role in mitochondrial calcium homeostasis by mediating mitochondrial calcium extrusion
		TPCN1	19'251'807 – 19'309’032	21'690'593 – 21'750’208	Thyroid gland disease, body mass index
		IQCD	19'312'105 – 19'338’590	21'750'459 – 21'776’876	Body mass index
		RITA1	19'340'169 – 19'345’131	21'778'451 – 21'783’438	Body mass index
		DDX54	19'344'935 – 19'364’848	21'783'672 – 21'803’059	Body mass index, believed to be involved in embryogenesis, spermatogenesis, and cellular growth and division
		CFAP73	19'364'941 – 19'372’019	21'803'025 – 21'810’252	Body mass index,

		RASAL1	19'380'566 – 19'407'774	21'818'249 – 21'845'715	Ossifying fibroma, juvenile ossifying fibroma, body mass index, visceral adipose tissue measurement, bone benign neoplasm, connective tissue benign neoplasm
		DTX1	19'408'742 – 19'414'443	21'846'683 – 21'882'543	Body mass index, cups of coffee per day measurement, visceral adipose tissue measurement, pthirus pubis infestation, lice infestation
Fetlock joint of the hind limb	27	*LOC106782668*	21'754'711- 21'779'842	21'801'757 – 21'827'111	-
		FRG1[a]	21'179'058 – 21'194'222	21'225'542 – 21'240'706	Fascioscapulohumeral muscular dystrophy 1, suppurative lymphadenitis, albuminuria, infant cerebrospinal fluid volume measurement, periodontitis
		TRIML1[a]	22'586'442 – 22'592'120	22'638'484 – 22644'276	Unknown function, associated with unipolar depression, response to selective serotonin reuptake inhibitor, polycystic ovary syndrome
Carpal joint	4	*CALCR*	37'066'892 – 37'131'479	37'113'944 – 37'258'137	Smoking behaviour, body mass index, physical activity measurement, hip circumference, birth weight, lean body mass, longitudinal BMI measurement, osteoporosis, bone mineral density quantitative trait locus 15 (osteoporosis, compression fractures (in homozygotes), metaphyseal fractures (in homozygotes))
		TFPI2	37'492'134 – 37'497'226	37'540'549 – 37'545'506	Resting heart rate, heart rate variability measurement, heart rate response to recovery post exercise, heart rate, fibrosarcoma, malignant ovarian cyst
		GNGT1	37'511'109 – 37'515'625	37'573'575 – 37'578'274	Resting heart rate, heart rate response to recovery post exercise, peripheral neuropathy, breast carcinoma, response to taxane, mosquito bite reaction size measurement, intelligence, Fructose-1,6-Bisphosphatase Deficiency, Retinitis Pigmentosa, Beta-Adrenergic

					Signaling, Development Dopamine D2 receptor transactivation of EGFR.
		GNG11	37’525’240 – 37’529’942	37’559’440 – 37’564’022	Resting heart rate, heart rate variability measurement, heart rate response to recovery post exercise, heart rate, mosquito bite reaction size measurement, beta-adrinergic signalling, Development Dopamine D2 receptor transactivation of EGFR
		BET1	37’601’073 – 37’611’154	37’649’405 – 37’659’486	Aging, aspartate aminotransferase measurement, serum alanine aminotransferase measurement, athletic endurance measurement, asparagine hypersensitivity, acute lymphoblastic leukemia
	3	*LCORL*	105’610’345 – 105’758’257	107’434’521 – 107’586’075	Body weight, birth weight, infant body height, heel bone mineral density, hip circumference, body mass index
		NCAPG	105’760’185 – 105’798’927	107’585’988 – 107’624’685	Body height, intelligence
		DCAF16	105’799’114 – 105’810’550	107’624’628 – 107’636’350	Body height, infant body height
		FAM184B	105’868’358 – 105’947’671	107’651’393 – 107’771’418	Body height, infant body height, parasitemia measurement, Trypanosoma cruzi seropositivity
		MED28	105’945’793 – 105’958’797	107’772’430 – 107’784’432	Infant body height
		LAP3	105’964’213 – 105’989’446	107’789’841 – 107’815’074	Bacterial Vaginosis, Nephronophthisis-Like Nephropathy 1
		CLRN2	106’026’205 – 106’037’711	107’851’793 – 107’863’374	Blood protein measurement
		QDPR	106’043’788 – 106’058’192	107’867’051 – 107’883’749	Blood protein measurement, reponse to carboplatine, Hyperphenylalaninemia Bh4-Deficient C, Phenylketonuria
	7	*SPATA19*	42’022’512 – 42’028’471	43’056’446 – 43’062’429	susceptibility to childhood ear infection measurement
		IGSF9B	42’067’916 – 42’123’524	43’097’490 – 43’157’010	Schizophrenia, intelligence, autism spectrum disorder, smoking status measurement, diastolic blood pressure, migraine disorder

		JAM3	42’275’597 – 42’293’136	43’261’488 – 43’326’636	Velopharyngeal dysfunction, schizophrenia, Hemorrhagic Destruction Of The Brain Subependymal Calcification and Cataracts
		NCAPD3	42’292’967 – 42’357’868	43’327’274 – 43’392’559	Microcephaly 22 Primary Autosomal Recessive (short stature, microcephaly, limb hypertonia, poor overall growth, normal to moderate developmental delay, seizures), Joubert Syndrome 8 (cerebellar ataxia, oculomotor apraxia, hypotonia, neonatal breathing abnormalities and psychomotor delay), word list delayed recall measurement, memory performance, neuropsychological test
		VPS26B	42’359’345 – 42’380’825	43’392’844 – 43’414’411	Late-Onset Parkinson Disease, word list delayed recall measurement, memory performance
		THYN1	42’381’262 – 42’386’993	43’414’848 – 43’420’597	-
		ACAD8	42’378’469 – 42’402’889	43’412’036 – 43’436’475	Isobutyryl-Coa Dehydrogenase Deficiency, Deficiency Of Short-Chain Acyl-Coa Dehydrogenase, fasting blood insulin measurement, amino acid measurement
		B3GAT1	42’666’260 – 42’696’496	43’701’213 – 43’731’984	Renal Adenoma, Natural Killer Cell Leukemia, N-Glycan measurement, prostate carcinoma, schizophrenia, alcohol drinking, word list delayed recall measurement, memory performance

[a] Surrounding genes closest to the QTL
