# Supplementary material for: Genome-Wide Association Studies Based on Equine Joint Angle Measurements Reveal New QTL Affecting the Conformation of Horses
Source: Genes (Basel). 2019 May 14;10(5):370. doi: 10.3390/genes10050370 (PMC6562990; doi:10.3390/genes10050370)
Supplement: Supplementary file 1 [file genes-10-00370-s001.zip › Table S2.pdf]

Table S2: Summary table of all GWAS

Trait	SNP name	ECA	Position EquCab 2	Position EquCab 3	Number of genotyped horses	Alleles (MAF) overall	Alleles (MAF) FM	Alleles (MAF) LIP	p-value
Poll	AX-103978374	28	12102163	13129283	495	0.2980	0.2095	0.4171	1.36E-07
Poll	AX-103396551	28	12101898	13129017	495	0.2949	0.2077	0.4123	1.44E-07
Poll	AX-103779310	1	124405158	125551151	495	0.1606	0.1514	0.1730	6.83E-07
Poll	AX-104440677	28	12106363	13133483	491	0.2301	0.1107	0.3886	8.36E-07
Elbow joint	AX-103649839	29	18799958	19878299	490	0.2327	0.1667	0.3199	1.69E-07
Carpal joint	AX-104691515	4	37412203	37460405	479	0.2630	0.1119	0.4550	6.07E-07
Carpal joint	AX-105008533	3	106128177	107955102	493	0.2241	0.0497	0.4573	6.24E-07
Carpal joint	AX-103944161	7	42659817	43694770	495	0.2323	0.1391	0.3578	8.83E-07
Fetlock joint of the fore limb	AX-104559840	23	49183107	48994679	494	0.0820	0.0813	0.0829	1.28E-05
Hip joint	AX-103391767	20	49183107	48994679	452	0.0697	0.1307	0.0000	6.10E-06
Stifle joint	AX-102974274	8	19266146	21704931	473	0.2918	0.1679	0.4455	2.77E-07
Hock joint	AX-103234126	21	30166547	31190691	490	0.2980	0.1900	0.4408	9.21E-06
Hock joint	AX-104124989	21	30263265	31287360	490	0.2980	0.1900	0.4408	9.21E-06
Fetlock joint of the hind limb	AX-103624054	27	22021462	22068848	495	0.1131	0.1338	0.0853	5.42E-07

P-value; P-value of the most significantly associated SNP per trait and locus corrected for genomic inflation (Pc1df from GenABEL)
